# Role of Hydrogen Sulfide in Early Blood-Brain Barrier Disruption following Transient Focal Cerebral Ischemia

**DOI:** 10.1371/journal.pone.0117982

**Published:** 2015-02-19

**Authors:** Zheng Jiang, Chun Li, Morganne L. Manuel, Shuai Yuan, Christopher G. Kevil, Kimberly D. McCarter, Wei Lu, Hong Sun

**Affiliations:** 1 Department of Cellular Biology & Anatomy, Louisiana State University Health Sciences Center-Shreveport, Shreveport, Louisiana, United States of America; 2 Department of Pathology, Louisiana State University Health Sciences Center-Shreveport, Shreveport, Louisiana, United States of America; University of Missouri, UNITED STATES

## Abstract

We determined the role of endogenous hydrogen sulfide (H₂S) in cerebral vasodilation/hyperemia and early BBB disruption following ischemic stroke. A cranial window was prepared over the left frontal, parietal and temporal cortex in mice. Transient focal cerebral Ischemia was induced by directly ligating the middle cerebral artery (MCA) for two hours. Regional vascular response and cerebral blood flow (CBF) during ischemia and reperfusion were measured in real time. Early BBB disruption was assessed by Evans Blue (EB) and sodium fluorescein (Na-F) extravasation at 3 hours of reperfusion. Topical treatment with DL-propargylglycine (PAG, an inhibitor for cystathionine γ-lyase (CSE)) and aspartate (ASP, inhibitor for cysteine aminotransferase/3-mercaptopyruvate sulfurtransferase (CAT/3-MST)), but not O-(Carboxymethyl)hydroxylamine hemihydrochloride (CHH, an inhibitor for cystathionine β-synthase (CBS)), abolished postischemic cerebral vasodilation/hyperemia and prevented EB and Na-F extravasation. CSE knockout (CSE^-/-^) reduced postischemic cerebral vasodilation/hyperemia but only inhibited Na-F extravasation. An upregulated CBS was found in cerebral cortex of CSE^-/-^ mice. Topical treatment with CHH didn’t further alter postischemic cerebral vasodilation/hyperemia, but prevented EB extravasation in CSE^-/-^ mice. In addition, L-cysteine-induced hydrogen sulfide (H_2_S) production similarly increased in ischemic side cerebral cortex of control and CSE^-/-^ mice. Our findings suggest that endogenous production of H_2_S by CSE and CAT/3-MST during reperfusion may be involved in postischemic cerebral vasodilation/hyperemia and play an important role in early BBB disruption following transient focal cerebral ischemia.

## Introduction

Ischemic stroke continues to be a leading cause of death and permanent disability worldwide [1]. Due to the advances in intravascular techniques and thrombolytic agents, transient focal cerebral ischemia has become one of the most common types of ischemic stroke. Although establishment of reperfusion is important for the cells in the penumbral zone, reperfusion is the most powerful independent predictor of BBB disruption [2], which can be found as early as within first hour of reperfusion [3]. Early BBB disruption has been considered as an antecedent event to infarction and hemorrhagic transformation [2,4]. A recent study found that patients with BBB disruption had a significantly reduced chance of major neurologic improvement and significantly higher risks of mortality and hemorrhagic complications after endovascular therapy [5]. To improve outcomes of patients with transient focal cerebral ischemia, it is important to develop therapeutic approaches against early BBB disruption.

H_2_S is a well-known toxic gas. Recent experimental studies have revealed that H_2_S is produced enzymatically in all mammalian species and serves as a gaseous signaling molecule involved in numerous biological processes. H_2_S can be endogenously generated from L-cysteine directly by enzymes, CBS, CSE and CAT/3-MST [6,7]. In addition, H_2_S is produced from d-cysteine through D-amino acid oxidase (DAO)/3MST in the cerebellum and kidney [8]. H_2_S has been demonstrated as a vasodilatory molecule with potent anti-inflammatory action in the cardiovascular system and referred as a neuromodulator and the third gasotransmitter in the central nervous system [9,10]. Unfortunately, only a few studies have investigated the role of H_2_S in the pathophysiology of ischemic stroke. A previous study found that high plasma L-cysteine is associated with poor clinical outcome in patients with ischemic stroke [11]. Furthermore, administration of L-cysteine dose-dependently increased the infarct volume in rat model of permanent focal cerebral ischemia [12]. On the other hand, exogenous H_2_S was recently reported to protect against global [13] and focal [14] cerebral ischemia/reperfusion injury. In addition, a protective effect of exogenous H_2_S on late BBB disruption was found in mouse model of transient focal cerebral ischemia [15]. As far as we are aware, no studies have investigated the possible role of endogenous H_2_S in early BBB disruption following ischemic stroke. Thus, our first goal was to determine whether endogenous H_2_S is involved in early BBB disruption following ischemic stroke. Reperfusion following transient focal cerebral ischemia can cause an increase in regional CBF (rCBF), hyperemia. Postischemic cerebral hyperemia occurs from vasodilation of the cerebral vasculature. Increasing evidence suggest that postischemic cerebral hyperemia associates with adverse events, including ischemic edema, BBB disruption, and poorer outcome [16,17]. Thus, our second goal was to determine whether endogenous H_2_S is related to postischemic cerebral vasodilation/hyperemia.

## Materials and Methods

### Preparation of animals

Animal studies were approved by the University Committee on Animal Resources of the Louisiana State University Health Science Center-Shreveport and conducted in accordance with the National Institute of Health Guide for the CARE and USE Laboratory Animals. CSE knock out (CSE^-/-^) mice on a C57BL/6J background were developed as described [18]. At 4 months of age (body weight 25 to 30 g), male C57BL/6J (n = 39) and CSE^-/-^ mice (n = 22) were anesthetized with thiobutabarbital sodium (Inactin, 100 mg/kg, ip), and a tracheotomy was performed. The mice were ventilated mechanically with room air and supplemental oxygen using a small animal ventilator (Harvard apparatus, March, Germany) at a fixed inhalation-exhalation ratio (1:1). A catheter was placed into right femoral vein for injection of Evans Blue (EB) and sodium fluorescein (Na-F). Blood pressure was measured using a CODA mouse tail-cuff system (Kent Scientific, Torrington, CT, USA). Body temperature was maintained at 37°C using a rectal temperature regulated heating pad (TC1000, CWE, Ardmore, PA, USA).

To perform ischemia/reperfusion, observe cerebral vasculature and topically administrate inhibitors of H_2_S producing enzymes, the mice were placed on a stereotaxic frame. A cranial window (6 mm x 8 mm, 1 mm from midline to the zygomatic arch) was prepared over the left frontal, parietal and temporal cortex. The cranial window was suffused with artificial cerebrospinal fluid bubbled continuously with 95% nitrogen and 5% carbon dioxide. The temperature of the suffusate was maintained at 37°C. The cranial window was connected via a three-way valve to an infusion pump, which allowed for infusion of inhibitors of H_2_S-producing enzymes into the cranial window. This method will maintain temperature, pH, PCO_2_ and PO_2_ of the cranial window at normal physiological range during the experiment. The MCA was ligated at its M2 segment just proximal to the first bifurcation/trifurcation with a 10–0 nylon suture for 2 hours. To prevent potential damage to the MCA during the ligation and easily induce reperfusion, a 0.25 mm length of 5–0 monofilament nylon suture was ligated together with the MCA. Reperfusion was induced by removing 5–0 nylon suture at 2 hours of ischemia. In Sham group, 10–0 nylon suture was not ligated.

### Experimental protocol

The cranial window was superfused with artificial cerebrospinal fluid for 30 minutes before ligating the MCA. We examined the influences of inhibitors of H_2_S-producing enzymes, (DL-propargylglycine (PAG) (an inhibitor for CSE), O-(Carboxymethyl)hydroxylamine (CHH) (an inhibitor for CBS) and aspartate (ASP) (an inhibitor for CAT)), on MCA ligation (MCAL)/reperfusion-induced regional vasodilation, hyperemia and early BBB disruption. The animals were divided into six groups: Control (n = 17), Sham (n = 4), PAG (n = 6), CHH (n = 6), ASP (n = 6), CSE^-/-^ (n = 16), and CSE^-/-^+CHH (n = 6). Drugs were mixed in artificial cerebrospinal fluid and then superfused into the cranial window. The superfusion of the inhibitors were started 10 minutes prior to the reperfusion and then continued throughout 3-hour reperfusion. The window concentration of PAG (100 μM), CHH (1 mM), and ASP (1 mM) are determined according to the previous reports [19,20,21,22].

### Measurement of cerebral vasodilation

Image recording of cerebral vasculature was started one minute before MCAL and continued throughout the experiment with an Orca-Flash 2.8 CMOS camera (Szxz-FOF, Olympus, Japan). The images at 5, 10, 30, 60, 90, and 120 minutes of ischemia and 5, 10, 30, 60, 90, 120, 150, and 180 minutes of reperfusion were analyzed with a Visiopharm Integrator System (Olympus, Japan). Percentage change of vascular diameter at the middle of the MCA branches, arterial anastomoses, and terminal branches of the anterior cerebral artery (ACA) and posterior cerebral artery (PCA), which are anastomosing the terminal branches of the MCA, were calculated. We classified all the imaged vessels by their baseline diameters: MCA: M3 (d ≥ 70 μm), M3 (60 μm ≤ d < 70μm), M3 (50 μm ≤ d <60 μm), M3 (40 μm ≤ d < 50 μm), M3 (30 μm ≤ d < 40 μm), M3 (d < 30 μm); A1 (terminal branches of ACA/PCA) and A2 (arterial anastomoses).

### Measurement of rCBF

To determine whether cerebral vasodilation during ischemia produces a hyperemia, we monitored rCBF at left somatosensory area. The probe of Laser Doppler Flowmetry (Periflux System 5000, Perimed, Sweden) was attached on the surface of parietal cortex 2 mm caudal and 4 mm lateral to the bregma. The rCBF was measured at the time points when cerebral vasculature was imaged. Changes in rCBF is calculated and expressed as percentage changes to the baseline.

### Assessment of BBB disruption

EB and Na-F were used to evaluate large and small solute permeability of BBB respectively. EB (4%, 6 ml/kg, Sigma) and Na-F (0.4%, 6 ml/kg, Sigma) saline solution were mixed and injected into the fermoral vein at two and half hours of reperfusion. At 3 hours of reperfusion, mice were transcardially punctured to collect blood sample and then perfuse saline (about 50 ml) until colorless fluid was obtained from the right atrium. Brains were removed quickly, frozen in liquid nitrogen and stored at −80°C. To determine the content of EB and Na-F in the brain tissues, ischemic side and contralateral cerebral hemispheres were separated, homogenized in 400 μl PBS and centrifuged at 4°C for 10 minutes at 1300 g. The supernatant was collected and protein concentration was determined by the Bradford method (Bio-Rad, CA, USA) with BSA as the standard. The supernatant was added with 50% trichloroacetic acid at 2:1 ratio and stored at 4°C for 12 hours. The mixture was centrifuged at 4°C for 15 minutes at 15000 g, 100μl supernatant was added with Borate buffer at 1:2 ratio and transferred to 96-well black plates. The concentration of Na-F was determined at 485-nm excitation/538-nm emission with CytoFluor Series 4000 fluorescence multiwell plate reader (PerSeptive Biosystems, MA, USA). To determine the content of EB in the brain tissues, the pellet of the homogenates were suspended with 500 μl formamide and incubated at 50°C for 72 hours. The mixture was centrifuged at 4°C for 20 minutes at 21000 g, and 300μl supernatant was transferred to 96-well black plate. The concentration of EB was determined at 550-nm excitation/620-nm emission with a Microplate Spectrophotometer, Spectra Max 190 (Molecular Devices, CA, USA). To normalize the content of EB and Na-F in brain tissues, plasma concentration of EB and Na-F was measured. Blood sample was centrifuged at 4°C for 10 minutes at 3000 g. The supernatant was diluted with water at 1:4 ratio. Diluted plasma was added with 20% trichloroacetic acid at 1:10 ratio and put into cool room (4°C) for 12 hours. The mixture was centrifuged at 4°C for 15 minutes at 15000 g, 100μl supernatant was added with Borate buffer at 1:2 ratio and transferred to 96-well black plates. The fluorescence of Na-F was read at 485-nm excitation/538-nm emission. To determine the content of EB in the blood samples, 25μl 1:4 diluted plasma was suspended with 400μl formamide and incubated at 50℃ for 72 hours. The mixture was centrifugation at 4°C for 20 minutes at 21000 g, and 30μl supernatant was transferred to 96-well black plate with 270 μl water for further dilution. The concentration of EB was read at 550-nm excitation/620-nm emission. The tissue content of EB and Na-F in ischemic side was normalized by its concentration in plasma and protein concentration of the homogenate and finally expressed as a ratio to its concentration in contralateral cerebral hemisphere.

### Western Blot Analysis

Additional twelve brains from the control mice (n = 6) with 2-hour ischemia/3-hour reperfusion and CSE^-/-^ mice (n = 6) were used to measure the expression of CSE, CBS and 3-MST. Under microscope, infarct core in control mice was identified as opaque area, and the cortex bordering 2 mm the infarct core was considered as the peri-infarct area. Parietal cortex tissues punched at the peri-infarct and contralateral corresponding areas of control mice or from CSE^-/-^ mice were used for measuring CSE, CBS and 3-MST. Brain tissues were homogenized in ice-cold lysis buffer containing 150 mmol/L NaCl, 50 mmol/L Tris HCl, 10 mmol/L EDTA, 0.1% Tween-20, 1%Triton, 0.1% mercaptoethanol, 0.1 mmol/L phenylmethyl sulfonylfluoride, 5 μg/mL leupeptin, and 5 μg/mL aprotinin, pH 7.4. Homogenates were centrifuged at 4°C for 10 minutes at 10000 g, and the supernatants were collected. Protein concentration was determined by the Bradford method (Bio-Rad, CA, USA) with BSA as the standard. SDS polyacrylamide gel electrophoresis (SDS-PAGE) was performed on a 10% gel on which 20 μg of total protein per well was loaded. After SDS-PAGE, the proteins were transferred onto polyvinylidene difluoride membrane. Immunoblotting was performed with the use of mouse anti-CSE (BD Bioscience, CA, USA), mouse anti-CBS (Santa Cruz, TX, USA), rabbit anti-3-MST (Santa Cruz, TX, USA) and rabbit anti-β-tubulin (Santa Cruz, TX, USA) as primary and peroxidase conjugated goat anti-mouse IgG and goat anti-rabbit IgG as the second antibodies. The bound antibody was detected by enhanced chemiluminescence (ECL) detection (Pierce Chemical, IL) and the bands were analyzed using ChemiDocTM MP Imaging System (Bio-Rad). For quantification, CSE, CBS and 3-MST proteins were normalized to the expressed β-tubulin.

### L-cysteine-induced H_2_S Production Assay

L-cysteine-induced H_2_S production was measured according to Bucci et al. with modifications [23]. Briefly, control (n = 5) and CSE knockout mice (n = 4) were sacrificed at 30 minutes of reperfusion. Ischemic side and contralateral cerebral cortex were isolated and homogenized in a lysis buffer (potassium phosphate buffer 100 mM pH = 7.4, sodium orthovanadate 10 mM and protease inhibitor cocktail (1:100 (v/v))). Homogenates were centrifuged at 4°C for 30 minutes at 15000 g. The supernatant was collected and protein concentration was determined by the Bradford method (Bio-Rad, CA, USA) with BSA as the standard. The supernatants (25 μl) were added in a reaction mixture (total volume 75 μl) containing piridoxal-5′-phosphate (5 mM, 10 μl), L-cysteine (100 mM, 4 μl), and saline (2 μl). The reaction was performed in parafilmed eppendorf tubes and initiated by transferring tubes from ice into an incubator (Fisher Scientific, MA, USA) at 37°C. After 30 minutes, ZnAc (0.85% in 3% NaOH, 100 μl) was added to trap evolved H_2_S. Subsequently, DPD (2 μM in 7.2 M HCl, 5 μl) and FeCl_3_ (30 μM in 1.2 M HCl, 6 μl) were added. After 20 min, absorbance values were read at a wavelength of 650 nm. H_2_S production was normalized with protein concentration and expressed as percentage change to control mice without I/R.

### Statistical Analysis

Data are reported as means ± SE. For comparison of the various treatments, results were analyzed with a one-way or two-way ANOVA as appropriate. Post hoc tests were performed using the Tukey’s test. Student t tests were used to compare CSE, CBS and 3-MST expression before and following transient focal cerebral ischemia and between control and CSE^-/-^ mice. A p value of 0.05 or less was considered to be significant.

## Results

### Control conditions

There is no significant difference in blood pressure between conscious control (96 ± 4/71 ± 2 mmHg) and CSE^-/-^ (99 ± 3/74 ± 2 mmHg) mice.

### MCAL/reperfusion-induced cerebral vasodilation

**Fig 1:**
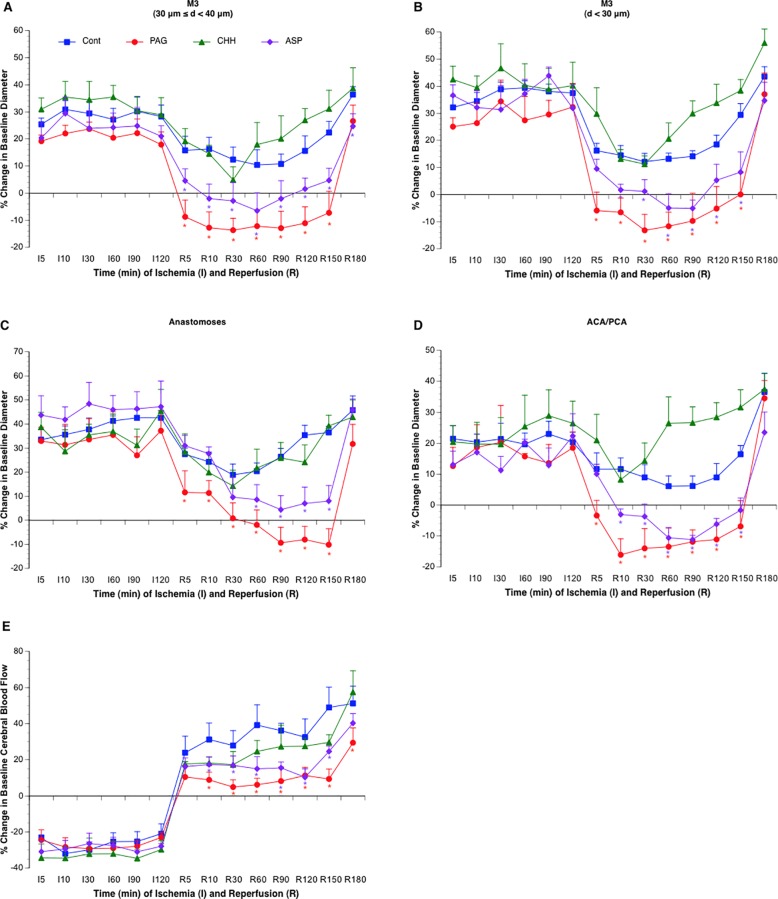
Effects of topical treatment with PAG (n = 6), CHH (n = 6), and ASP (n = 6) on vasodilation of the MCA (A-B), arterial anastomoses (C) and terminal branches of the ACA/PCA (D) and CBF of somatosensory area (E) during 2-hour MCAL/3-hour reperfusion. Values are means ± SE. *P < 0.05 vs. Control.

Cerebral vasodilation started immediately after MCAL and continued during the entire 2-hour ischemia and 3-hour reperfusion in nearly all branches of MCA, arterial anastomoses, and terminal branches of ACA/PCA. The magnitude of vasodilation was varied in arteries more than 40 μm in diameter and stable in arteries equal or less than 40 μm in diameter. In addition, the rCBF increased during the entire 3-hour reperfusion (Fig. 1).

### Effects of inhibitors of H_2_S-producing enzymes on postischemic cerebral vasodilation and rCBF

As shown in Fig. 1, topical treatment with 100 μM PAG and 1 mM ASP abolished cerebral vasodilation in all branches of the MCA, arterial anastomoses and terminal branches of ACA/PCA during reperfusion. Conformably, the rCBF was significantly reduced in PAG- and ASP-treated groups. In contrast, topical treatment with 1 mM CHH failed to attenuate the cerebral vasodilation and postischemic hyperemia.

### Effects of inhibitors of H_2_S-producing enzymes on BBB disruption

**Fig 2:**
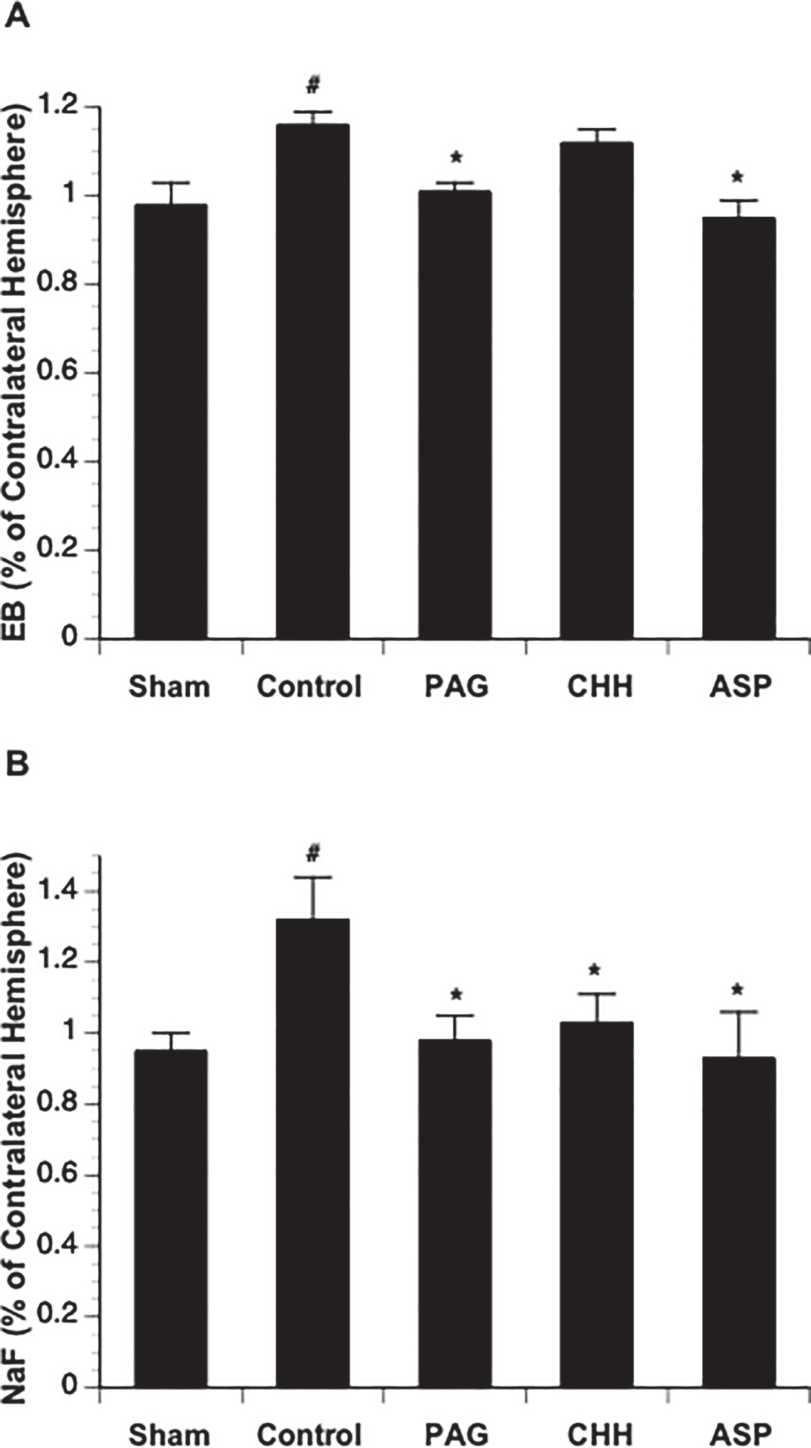
Effects of topical treatment with PAG (n = 6), CHH (n = 6), and ASP (n = 6) on EB (A) and Na-F (B) extravasation at 3 hours of reperfusion. Values are means ± SE. *P < 0.05 vs. Control.

Since the ligation of the MCA in our new model was complete with little individual variability, the extravasation of EB and Na-F was steady and significant at 3 hours of reperfusion in mice undergone MCAL compared with the mice in Sham group (Fig. 2). Topical treatment with PAG and ASP completely prevented both EB and Na-F extravasations at 3 hours of reperfusion. In contrast, topical treatment with CHH only significantly attenuated Na-F extravasation (Fig. 2).

### Cerebral vasodilation, rCBF and BBB disruption in CSE^-/-^ mice

**Fig 3:**
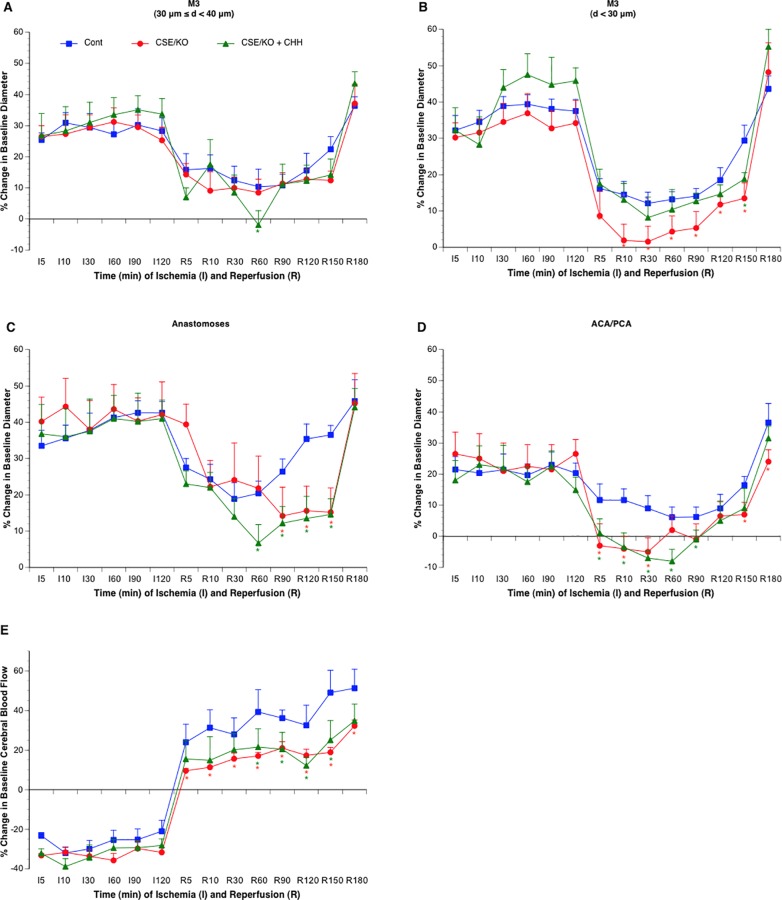
Effects of CSE knockout with (n = 6) or without (n = 6) CHH treatment on vasodilation of the MCA (A-B), arterial anastomoses (C) and terminal branches of the ACA/PCA (D) and CBF of somatosensory area (E) during 2-hour MCAL/3-hour reperfusion. Values are means ± SE. *P < 0.05 vs. Control.

**Fig 4:**
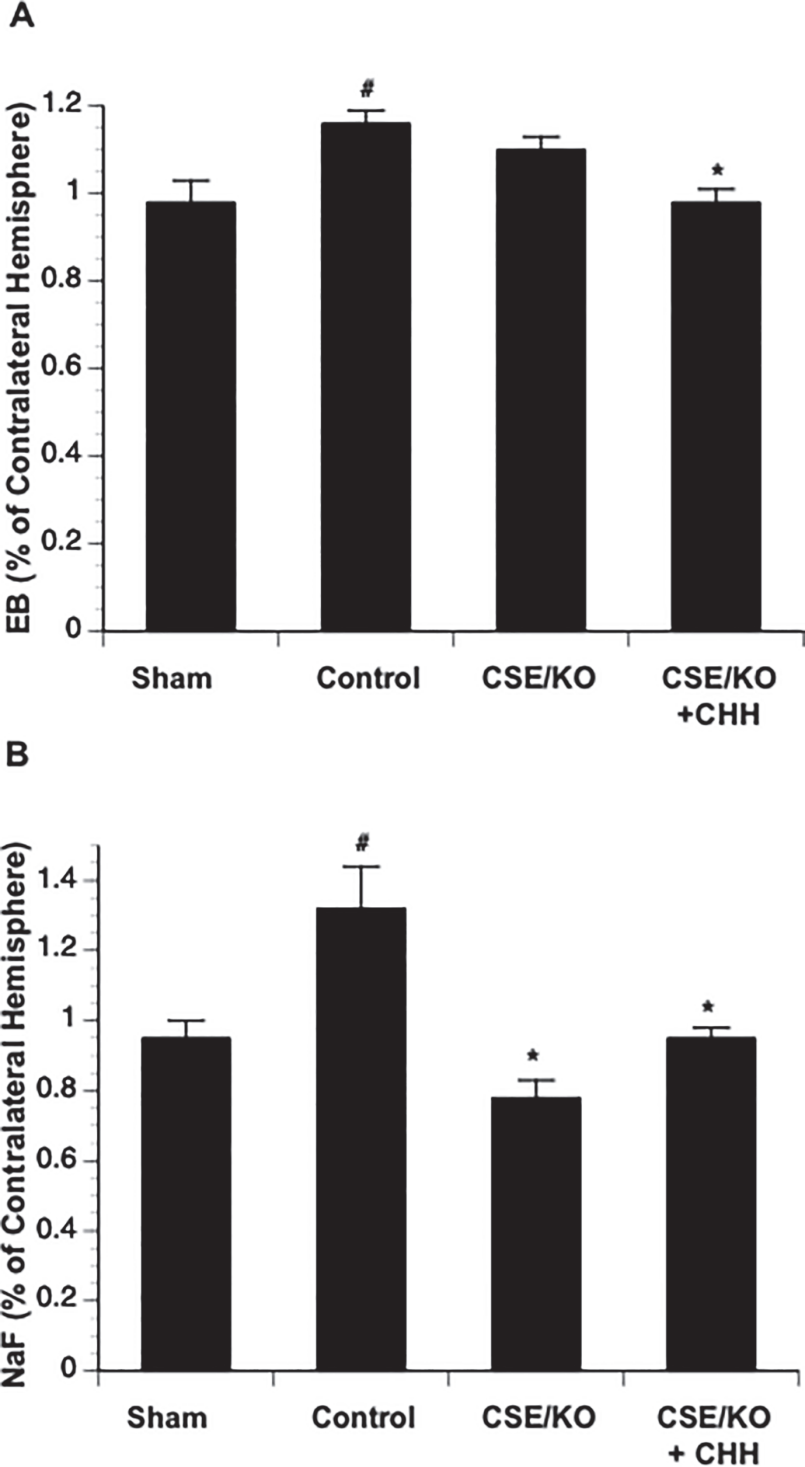
Effects of CSE knockout with (n = 6) or without (n = 6) CHH treatment on EB (A) and Na-F (B) extravasation at 3 hours of reperfusion. Values are means ± SE for 6 mice in each group. *P < 0.05 vs. Control.

**Fig 5:**
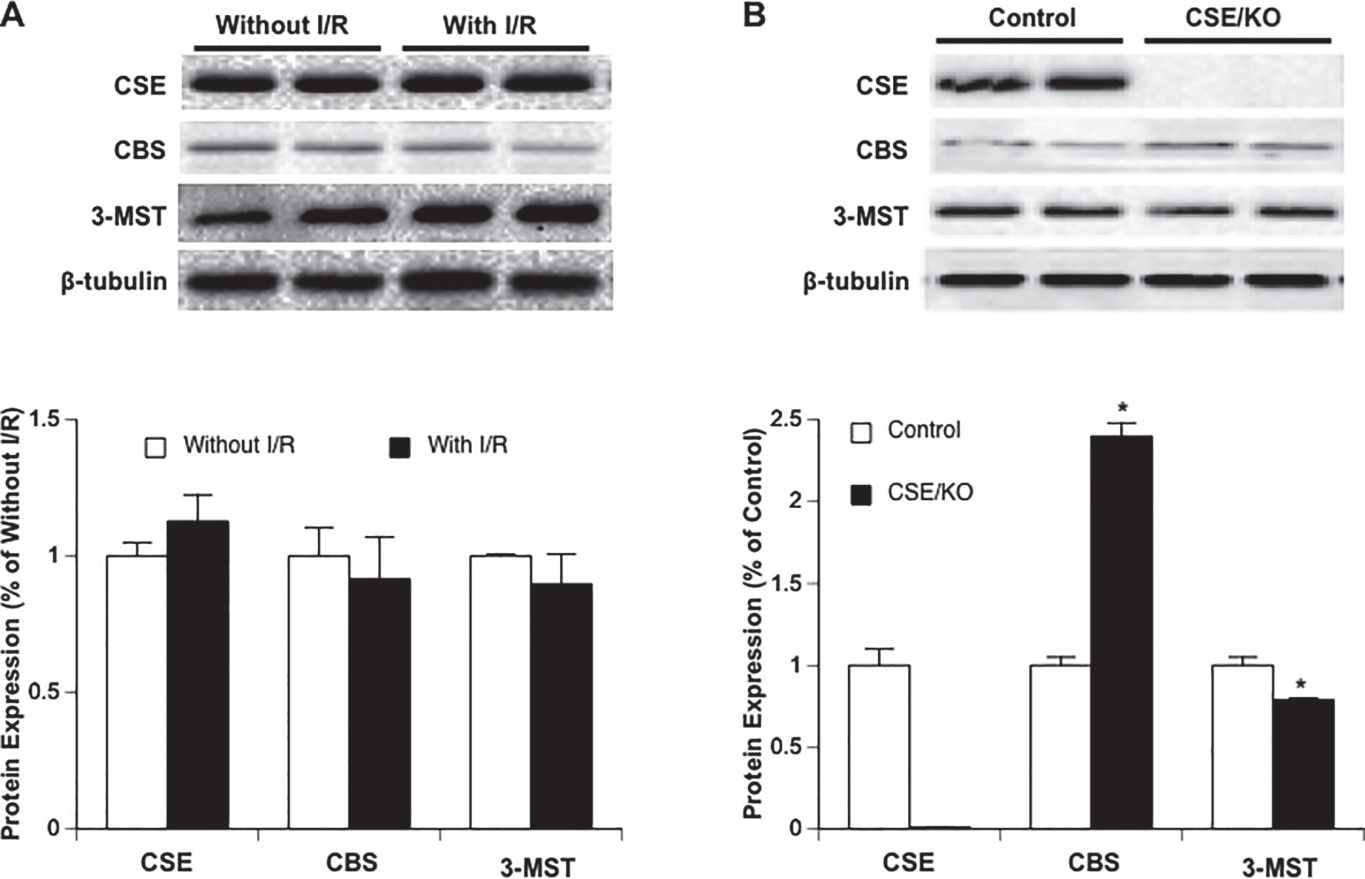
(A) Effect of 2-hour MCAL/3-hour reperfusion on protein expression of CSE, CBS and 3-MST in parietal cortex of control mice (n = 6). (B) Protein expression of CSE, CBS and 3-MST in parietal cortex of CSE^-/-^ mice (n = 6). Values are means ± SE. *P < 0.05 vs. Control.

There was no difference in baseline diameter between control and CSE^-/-^ mice. Cerebral vasodilation and rCBF significantly reduced during MCAL in CSE^-/-^ mice compared to the control mice. During reperfusion, although CSE knockout only significantly reduced cerebral vasodilation in arterial anastomoses, a reduced rCBF was still observed in CSE^-/-^ mice (Fig. 3). Surprisingly, CSE knockout only produced an inhibition in Na-F extravasation but not EB extravasation (Fig. 4). Thus, we measured protein expression of CBS and 3-MST in parietal cortex of CSE^-/-^ mice. Interestingly, CBS expression upregulated more than twofold, whereas 3-MST expression slightly downregulated in CSE^-/-^ mice (Fig. 5). Thus, we further examine the effect of CBS inhibitor on cerebral vasodilation, rCBF and BBB disruption in CSE^-/-^ mice. As shown in Figs. 3 and 4, topical treatment with CHH didn’t further reduce cerebral vasodilation and rCBF, but completely prevented EB extravasation as well as Na-F extravasation.

### Expression of H_2_S-producing enzymes following 2-hour MCAL/3-hour reperfusion

To determine whether postischemic hyperemia and early BBB disruption is related to an increased expression in H_2_S-producing enzymes, we measured the protein expression of CSE, CBS and 3-MST in parietal cortex punched at the peri-infarct area. As shown in Fig. 5, all three H_2_S-producing enzymes were not altered at 3 hours of reperfusion.

### L-cysteine-induced H_2_S production

**Fig 6:**
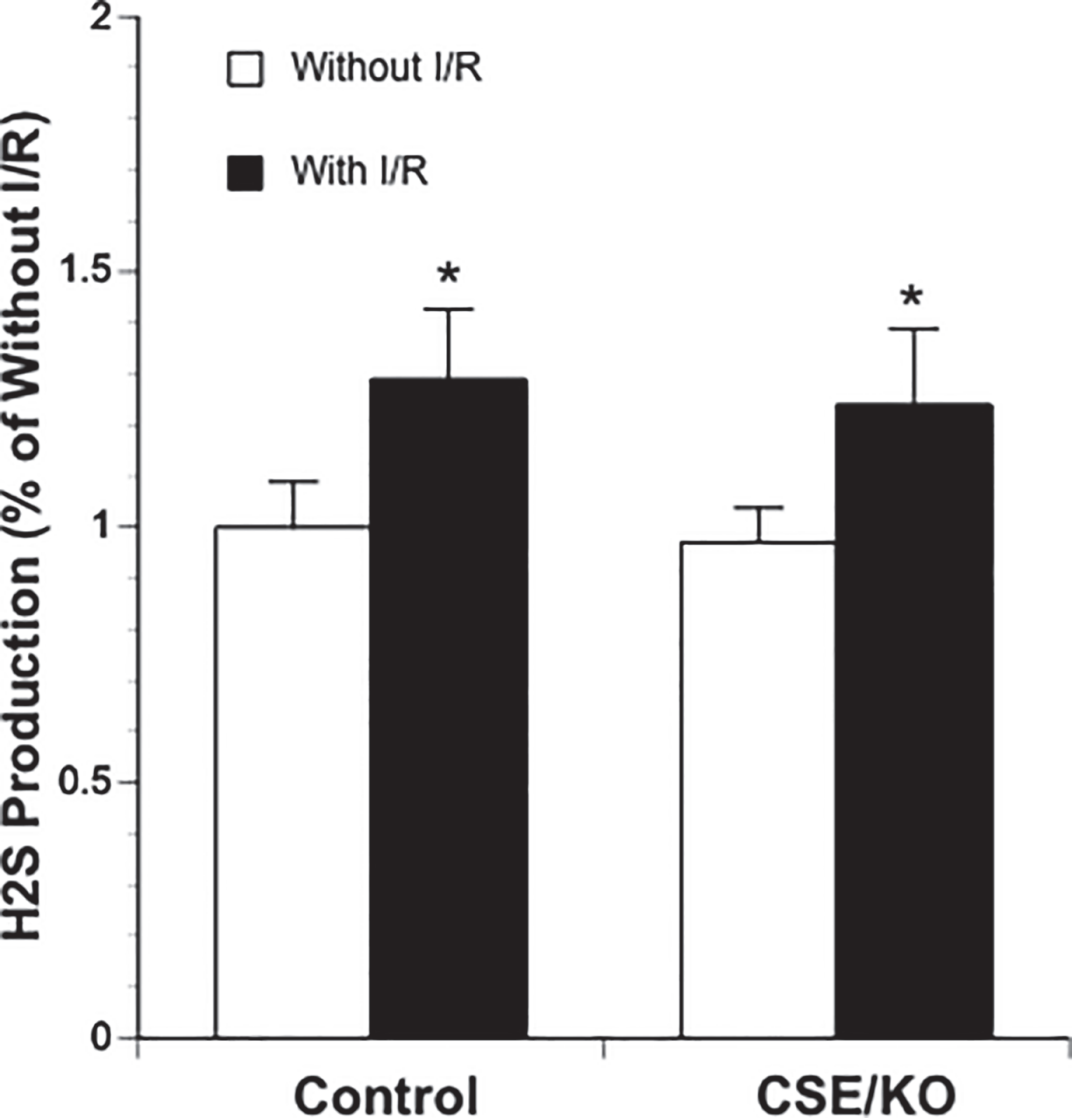
L-cysteine-induced H_2_S production in ischemic side and contralateral cerebral cortex of control (n = 5) and CSE knockout mice (n = 4). Values are means ± SE. *P < 0.05 vs. Without I/R.

To determine whether postischemic hyperemia and early BBB disruption is related to an increased activity in H_2_S-producing enzymes, we measured L-cysteine-induced H_2_S production in ischemic side and contralateral cerebral cortex. As shown in Fig. 6, L-cysteine-induced H_2_S production significantly increased in ischemic side cerebral cortex of both control and CSE^-/-^ mice. There was no significant difference in L-cysteine-induced H_2_S production between control and CSE^-/-^ mice.

## Discussion

The present study first investigated the role of H_2_S in postischemic cerebral vasodilation/hyperemia and early BBB disruption. There are several new findings from this study. First, topical treatment with inhibitors of CSE and CAT/3-MST significantly reduced postischemic cerebral vasodilation/hyperemia and completely prevented early Na-F and EB extravasation following transient focal cerebral ischemia. Second, an upregulated CBS was found in cerebral cortex of CSE^-/-^ mice. Although CBS inhibitor didn’t alter postischemic cerebral vasodilation/hyperemia, it further prevented early EB extravasation in CSE^-/-^ mice. Third, L-cysteine-induced H_2_S production significantly increased in ischemic side cerebral cortex of both control and CSE^-/-^ mice. Our findings suggest that CSE- and CAT/3-MST-mediated H_2_S production may play a critical role in cerebral vasodilation/hyperemia and early BBB disruption following transient focal cerebral ischemia. In addition, postischemic cerebral vasodilation/hyperemia may not associate with early BBB disruption.

CBS, CSE and CAT/3-MST are considered as major enzymes producing H_2_S in the brain under physiological conditions. However, these enzymes have different cellular distributions. CBS is strongly expressed in the hippocampus and cerebellum and weakly expressed in the neurons of the cerebral cortex, striatum and thalamus [24]. CSE is mainly found in vascular endothelial cells and smooth muscle cells [18,25]. In contrast, 3-MST has been reported to express in vascular endothelial cells [26], astrocytes [27] and neurons [28]. In the present study, topical treatment with PAG and ASP produced a strong inhibitory effect on cerebral vasodilation and rCBF during early reperfusion. In addition, PAG and ASP completely inhibited early Na-F and EB extravasation. PAG is a selective CSE inhibitor [29] and ASP is competitive substrate for CAT [22]. Thus, our findings suggest that CSE and 3-MST may be involved in postischemic cerebral vasodilation/hyperemia and early BBB disruption. On the other hand, CHH, topical given at 1 mM, only significantly inhibited early Na-F extravasation, failed to produce any inhibitory effect on cerebral vasodilation and rCBF. The absence of inhibitory effect of CHH is not likely related to the approach, dose and duration of the treatment. In a recent study, topical treatment with neuronal nitric oxide synthase (nNOS) inhibitors, N(omega)-propyl-L-arginine (L-NPA) and 7-nitroindazole (7-NI), significantly inhibited neuronal nitric oxide synthase (nNOS) [30]. Furthermore, topical treatment with CHH at 1 mM produced a potent inhibitory effect on EB extravasation in CSE^-/-^ mice. Thus, CBS may not play a major role in the postischemic cerebral vasodilation/hyperemia and early BBB disruption.

The vasodilatory effect of H_2_S has been reported in several different kinds of arteries, including thoracic aorta [31], mesenteric [32], pulmonary [33], hepatic [34], and tail arteries [25]. The present study further found that H_2_S is also involved in the vasodilation of cerebral arteries. Generally, H_2_S relaxes vascular smooth muscle by opening ATP-dependent K^+^ channels in a non-ATP-associated manner [35]. However, the vasodilatory effect of H_2_S alone is weak. A synergistic effect between H_2_S and nitric oxide (NO) may exist [31]. NO donor was shown to increase H_2_S production by upregulating CSE expression/activity [25]. Furthermore, H_2_S-induced vasodilation was attenuated either by removal of endothelium or by blockage of NOS, suggesting that H_2_S may promote endothelial cells to release NO [36]. It has been suggested that a burst of NO generation occurs within the first few minutes after ischemia [37,38]. On the other hand, the endogenous level of H_2_S in the affected cerebral cortex significantly increased following ischemic stroke [12]. In the present study, CSE and 3-MST inhibitors nearly completely abolished the cerebral vasodilation during reperfusion. Interestingly, topical treatment with NOS inhibitor similarly abolished cerebral vasodilation in a recent study [30]. Thus, postischemic cerebral vasodilation/hyperemia may be mediated by an interaction between NO and H_2_S.

Hyperemia during reperfusion was reported to associate with adverse events including BBB disruption in rat MCAO model [16]. Excessive production of H_2_S and NO might lead BBB disruption via a sustained hyperemia during reperfusion. In the present study, however, although CHH failed to inhibit cerebral vasodilation/hyperemia during reperfusion, it prevented early BBB disruption in CSE^-/-^ mice. In a previous study, we found that papaverine abolished the inhibitory effect of L-NAME on cerebral vasodilation/hyperemia, but preserved the inhibitory effect of L-NAME on early BBB disruption. In addition, an increased hyperemia was concurrently found with a reduced BBB disruption in a group topically treated with papaverine alone [30]. These findings suggest that excessive production of H_2_S and NO during reperfusion may lead to BBB disruption via a vasodilation/hyperemia-independent mechanism. Since both early BBB disruption and postischemic hyperemia may associate with an excessive production of H_2_S and NO, it was not surprised to observe an illusive correlation between hyperemia and early BBB disruption [16]. In fact, hyperemia during early reperfusion may be beneficial for preventing early BBB disruption.

There are several potential mechanisms that may account for the detrimental effect of H_2_S on BBB integrity. First, overproduction of H_2_S may lead endothelial cell damage by arresting aerobic metabolism via direct inhibition on mitochondrial cytochrome oxidase. Second, overproduction of H_2_S may increase S-sulfhydration level of tight junction (TJ) and/or adherens junction (AJ) proteins and thus alter their function. Third, overproduction of H_2_S may promote early BBB disruption via NMDA receptor-mediated excitotoxicity of glutamate. Previous studies have shown that H_2_S potentiates NMDA receptor function [10] and blockade of NMDA receptor significantly attenuated early BBB disruption following focal cerebral ischemia [39]. Fourth, overproduction of H_2_S may induce early BBB disruption via enhancing NO release. As described above, H_2_S promotes endothelial cells to release NO [36]. Overproduction of NO may lead endothelial cell damage by directly altering protein structure/function and/or indirectly through the formation of highly reactive peroxynitrite. The rapid restoration of blood flow following ischemia produces a burst of superoxide generation, which result in a rapid increase in peroxynitrite formation. Peroxynitrite has been demonstrated to activate matrix metalloproteinases (MMPs) 2 and 9, which are two major enzymes responsible for BBB disruption, cerebral edema, hemorrhagic transformation and progressive inflammatory reactions following transient focal cerebral ischemia.

A few studies have experimentally investigated the role of H_2_S in the pathophysiology of ischemic stroke. Qu et al showed that administration of L-cysteine dose-dependently increased the infarct volume in rat model of permanent focal cerebral ischemia [12]. In contrast, systematic treatment of H_2_S donor, NaHS, was recently found to protect against global [13] and focal [14] cerebral ischemia/reperfusion injury. In these studies, however, NaHS was systemically given at the onset of ischemia. Thus, the neuroprotective effect might be related to the vasodilatory effect of exogenous H_2_S on arterial anastomoses and terminal branches of unoccluded cerebral arteries. More recently, H_2_S donors, given at 3 hours of reperfusion, were shown to significantly prevent late BBB disruption [15]. In the present study, however, CSE and CAT/3-MST inhibitors, topically given at the beginning of reperfusion, completely abolished early BBB disruption. Thus, it would be necessary to further evaluate the effect of CSE and CAT/3-MST inhibitors on late BBB disruption.

In summary, the present study determined the role of endogenous H_2_S in postischemic cerebral vasodilation/hyperemia and early BBB disruption following transient focal cerebral ischemia. Our findings suggest that endogenous H_2_S production is involved in postischemic cerebral vasodilation/hyperemia and early BBB disruption. Although H_2_S-mediated cerebral vasodilation/hyperemia didn’t appear to relate to early BBB disruption, CSE and 3-MST inhibitors significantly prevented early BBB disruption following transient focal cerebral ischemia.
